# MEDICAL STUDENTS’ CONFIDENCE INTERVIEWING, AND PERCEPTIONS OF, OLDER ADULTS

**DOI:** 10.1093/geroni/igae098.2698

**Published:** 2024-12-31

**Authors:** Aliza Baron, Seema Limaye, Stacie Levine, Monica Long, Shellie Williams

**Affiliations:** University of Chicago, Chicago, Illinois, United States; Edward Hines, Jr. VA Hospital, Oak Park, Illinois, United States; University of Chicago, Chicago, Illinois, United States; University of Chicago Medicine/SHARE Network, Glenwood, Illinois, United States; University of Chicago, Chicago, Illinois, United States

## Abstract

As the U.S. populations ages, medical students need primary geriatric skills yet continue to lack interest in geriatric care and hold negative attitudes towards caring for older adults (OA). The Geriatrics and Aging Through Transitional Environments (GATE) Curriculum for first year Pritzker medical students provides experiential learning conducting a functional history interview and assessing frailty and home safety risks. Students attended a 1-hour lecture on geriatric functional history taking, frailty (fatigue, resistance, weight loss, gait, hospitalization) and home safety assessments. Student pairs conducted an in-home structured interview with an OA “trained patient” (TP) or untrained OA family/acquaintance. OA residents at a local retirement community were recruited and trained as TP through a 1-hour training session on-site. Training addressed students’ learning objectives, interview questions, and techniques for delivering feedback to students. Learner evaluation included a pre/post survey assessing self-confidence interviewing OA using a Likert scale (5=Extremely confident, 1=Not at all) and attitudes towards OA using open-ended word association. From 2021-23, 178 Pritzker medical students (100%) completed 96 interviews (86% with TP, 14% with family/acquaintances) with 55 OA. Confidence interviewing changed significantly pre-GATE (M=1.73, SD=.720) to post-GATE (M=3.43, SD=.814); t(168)=-23.88, p<.001. Students’ attitudes towards OA shifted post-GATE with an increased usage of word associations “wise/knowledgeable” and “storytellers” and decreased associations as “frail, slow, diseased.” A medical student curriculum incorporating in-home functional history taking interviews with OA TP may increase students’ confidence interviewing and instill positive attitudes towards OA that are foundational for building empathic doctor-patient relationships across medical specialties.

